# Exploring the Entrepreneurial Intention-Competency Model for Nascent Entrepreneurs: Insights From a Developing Country Context

**DOI:** 10.3389/fpsyg.2021.516120

**Published:** 2021-07-28

**Authors:** Melodi Botha, Amorie Taljaard

**Affiliations:** Department of Business Management, University of Pretoria, Pretoria, South Africa

**Keywords:** entrepreneurial competencies, entrepreneurial intention, developing country context, higher order competence constructs, cognitive and functional competence

## Abstract

Several previous scholars have investigated the relationship between entrepreneurial intention (EI) and entrepreneurial competencies (ECs), yet categorising individual ECs in relation to higher-order competence constructs has not been explored. Based on the previous literature studies, four higher-order constructs are identified, namely cognitive, functional, social/personal and meta-competence. Investigating which ECs are categorised according to the four higher-order constructs in this relationship with EI is important as it contributes to the development and training of these antecedents of entrepreneurial behaviour. Data are collected from 203 nascent entrepreneurs in South Africa and analysed by using structural equation modelling. In this developing country context, only two higher-order constructs, cognitive/functional competence and social/personal competence, fit the data in relationship with EI. The strongest positive relationships were found between the cognitive and functional higher-order construct, opportunity recognition and opportunity assessment, conveying a compelling vision and creative problem-solving. The cognitive and functional higher-order competence construct also showed a strong positive relationship with EI. To improve pedagogical interventions and enhance EI, educators and policymakers can use these findings in entrepreneurship programmes to ensure that this higher-order competence construct with the individual ECs in this category are developed simultaneously rather than individually. Research efforts and support programmes that include cognitive and functional higher-order competence constructs for nascent entrepreneurs should therefore not be neglected. Such efforts and programmes can encourage EI, which in turn can enhance entrepreneurial behaviour, thereby potentially contributes to economic growth and employment creation.

## Introduction

Entrepreneurship is widely argued to be critical for alleviating poverty and unemployment. Pendame (2014) stresses that the development of entrepreneurial intention (EI) and the creation of new businesses are of utmost importance for economic advancement. This is even more important in the context of a developing country, for example, South Africa with its alarming high unemployment rate of 29.1% in 2019 (Trading Economics, 2019). Despite the knowledge of its benefits, EI in South Africa has decreased significantly in 2017 relative to 2013 (from 15.4 to 11.7%), which is substantially lower than Africa as a whole, and half of what the efficiency-driven economies achieve (Herrington and Kew, 2018). A possible explanation for this could lie in factors, such as a lack of education, corruption and a restricting regulatory environment (Urban, 2012), which negatively affect the number of intentional entrepreneurs (Herrington and Kew, 2018). As an efficiency-driven economy, South Africa has been identified as one of the least supportive countries constraining entrepreneurship, based on the entrepreneurial framework conditions set out in the Global Entrepreneurship Monitor (GEM) report, with an average (AVE) rating of 4.0 for 8 out of 12 conditions (Herrington and Kew, 2018). Thompson (2009) advances that EI is most practically defined as a self-acknowledged persuasion by a person to set up a new business venture at some points in the future. Although Santos et al. (2010) state that EI enables the prediction of innovation behaviour, motivation and acting as a guide towards action, a previous work on EI highlighted that the start-up rate of businesses by these individuals is very low while the EI of nascent entrepreneurs may be high (Nabi et al., 2010; Singh et al., 2016). The most popular samples that previous studies have measured EI on are student samples (i.e., Boyd and Vozikis, 1994; Thompson, 2009; Prodan and Drnovsek, 2010; Arasti et al., 2012). Similarly, these scholars call for research investigating other samples and specifically antecedents that have positive relationships with EI. There is evidence that the self-efficacy and other entrepreneurial competencies (ECs) of nascent entrepreneurs correlate highly with EI, which enhances entrepreneurial behaviour (Clercq and Arenius, 2006; Hsu et al., 2017).

One way of understanding the behaviour of nascent entrepreneurs is by studying their EC levels, e.g., their skills, knowledge and attitudes (Mitchelmore and Rowley, 2010). Because competency refers to the skills, knowledge, attitudes and quality of action taken by entrepreneurs, Bird (1995) and Morris et al. (2013) suggest that it is directly related to entrepreneurial action (start-up). ECs also received attention in preceding the entrepreneurship literature and many scholars identified a core set of ECs that are crucial for inclusion when developing entrepreneurs (Man et al., 2002; Clercq and Arenius, 2006; Mitchelmore and Rowley, 2010; Morris et al., 2013; Hsu et al., 2017). Therefore, ECs have been identified as an explicit set of competencies relevant to the implementation of successful entrepreneurship, which is often correlated with the intention and action of developing a business (Bird, 1995; Mitchelmore and Rowley, 2010). Rasmussen et al. (2011) argue that there is a gap in the literature relating to how competencies are categorised, and which ones are related to different competence categories. Previous scholars have focused on predicting the skills necessary to start a business and have conducted studies on students or pre-entrepreneurs and nascent entrepreneurs (Zhao et al., 2010; Morris et al., 2013). We acknowledge the belief of Morris et al. (2013) that the right set of ECs can enhance EI and even more so entrepreneurial action. Scholars such as Baum et al. (2001), Bird (1995), Colombo and Grilli (2005) and Mitchelmore and Rowley (2010) have confirmed that different ECs are learnt and necessary at different stages of the venture life cycle. Winterton et al. (2006) categorised competence in four dimensions. Skills are captured by functional competence, knowledge is captured by cognitive competence, attitudes and behaviours are captured by social competence while meta-competence, which is rather different from the first three dimensions, is concerned with facilitating the acquisition of the other substantive competence (Winterton et al., 2006:41). In this paper, we adopt this typology by categorising the individual ECs in relation to these higher-order constructs.

Multiple studies have measured EI among students and graduates (Hayton et al., 2002; Turker and Sonmez Selçuk, 2009; García-Rodríguez et al., 2015), yet there is a lack of research measuring the relationship between EI and ECs, specifically in a developing country context on samples other than students (Clercq and Arenius, 2006; Fatoki, 2010; Hsu et al., 2017). Previous studies, conducted in developed countries, have modelled EI (Thompson, 2009) and EC (Mitchelmore and Rowley, 2010) as outcomes in separate models. There is evidence suggesting that EI and ECs can be condensed into one model (Al Mamun et al., 2016). The social cognitive theory (SCT), as proposed by Bandura (1986), provides an underlying framework suggesting that EI and ECs can influence each other, inferring that a bidirectional relationship is possible. This view is supported by Thompson (2009) who suggests that EI can also be treated as an independent variable, indicating that EI can predict certain outcomes. The social (Coleman, 1988) and human capital theories (Becker, 1993) further support the relationship of EI and ECs and the strength of the model fit when examining nascent entrepreneurs.

The purpose of this paper is to investigate the EI–ECs model from an individual-level approach, and the model is tested to explore whether ECs are related to higher-order constructs [by splitting them into categories as suggested by Winterton et al. (2006)] in their relationship with EI. Structural equation modelling (SEM) is conducted on 203 nascent entrepreneurs in South Africa, and correlation was used to test the strength of the relationships between EI, the individual ECs and the higher-order competence constructs. An EI–EC model with two higher-order competence constructs, namely cognitive and functional as well as social/personal competence, indicated the most acceptable and parsimonious model fit. Cognitive and functional higher-order competence proved to have a strong positive relationship with EI, whereas social/personal higher-order competence had a negative weak relationship with EI. Therefore, cognitive and functional higher-order constructs with the individual ECs categorised under this construct should be developed simultaneously to enhance the EI levels of nascent entrepreneurs. These findings have implications for theory and practise. Firstly, from a theoretical viewpoint, investigating the relationships between EI and the higher-order competence constructs with individual ECs categorised under each construct, have merits as it has received scant research attention to date. Secondly, the findings in this paper contribute to the body of knowledge, particularly the SCT, theory of planned behaviour (TPB) as well as human and social capital theories, in an entrepreneurial context. From a practical viewpoint, the findings regarding the strength of the relationships may guide policy interventionists and educators to focus on the most impactful higher-order competence constructs such as cognitive and functional and their complex relationship with the individual ECs categorised under this construct and EI. This can augment pedagogical interventions as well as spawn entrepreneurial action for nascent entrepreneurs. In particular, interesting insights into the relationship of EI and the cognitive and functional higher-order competence construct are brought to light, which may be valuable for such interventions. A further contribution of this paper lies in the geographical sphere. Because this research is conducted in South Africa, we answer the call for entrepreneurship research in an African context (George et al., 2016), specifically, for EI research in Africa (García-Rodríguez et al., 2015). This research carries far broader applicability for developed economies while remaining relevant to other developing countries with similar entrepreneurial activity levels. Many developed country research studies (i.e., Boyd and Vozikis, 1994; Hayton et al., 2002; Thompson, 2009; Turker and Sonmez Selçuk, 2009; Prodan and Drnovsek, 2010; Arasti et al., 2012; García-Rodríguez et al., 2015) concentrated on student samples, whereas this paper focused on a nascent entrepreneur sample.

The paper commences with a theoretical foundation on the relationship between EI and ECs, more specifically focusing on existing theories to provide a supportive framework. A set of interrelated ECs as well as categorising ECs into higher-order competence constructs are discussed and hypotheses are presented. Afterwards, the methodology, procedure, measures and results are presented, followed by hypothesis testing and discussion of the results, concluding remarks and limitations and recommendations for future research areas.

## Theoretical Foundation and Hypotheses Development

### EI Research in Developed and Developing Countries

Research on EI stems from the TPB as proposed by Ajzen (1991), who states that attitude towards behaviour, subjective norm and perceived behavioural control are the three factors that precede any type of planned behaviour. Bird (1988) and Boyd and Vozikis (1994) advance the theory that intention is a state of mind that centres a person's attention, experience and behaviour towards a specific method of behaving. It is suggested that EI motivates critical strategic thinking and resolutions, and functions as a perceptual monitor for observing relationships, resources and exchanges. It also offers a means to more effectively describe and predict entrepreneurship (Krueger et al., 2000). While EI research is lacking in developing countries (García-Rodríguez et al., 2015), a few literature studies are available with a specific reference to the difference in EI between a developing and developed country (Iakovleva et al., 2011). One study found that Norway (the developed country in this case) had a lower EI among students in comparison to Indonesia. This was attributed to the economic and social status exhibited by Norway (Kristiansen and Indarti, 2004). Yet another study on graduate EI in Malaysia identified that EI is affected by the environment and the support that the potential entrepreneur is likely to receive in the country they operate in (Trivedi, 2017). The environment and support are factors which differ between developed and developing countries. Moreover, only 17% of all start-ups are driven by necessity in developed economies, vs. 32% in developing countries (Bosma and Levie, 2009). Consequently, we acknowledge a distinction between developing countries such as South Africa and Brazil, and developed countries such as Australia and Germany (World Economic Forum, 2017), where the developing countries have much lower EI and entrepreneurial action levels (Singer et al., 2018). However, based on the Global Entrepreneurship Monitor (GEM) report (Herrington et al., 2019), in contradiction to the World Economic Forum, research results indicate that 22 out of 48 countries identified as developing countries have a much higher EI rate than developed countries, indicating an AVE of 33.8 vs. 15.1, and a TEA AVE rate of 16.9 for developing countries and 9.3 for developed countries. However, based on going from intentions to actual entrepreneurial activity, the gap between developing countries is bigger than that between developed countries. On average, almost half of the entrepreneurs in developing countries with EI do not go over to action. Many factors can play a role such as income levels, ease of doing business, entrepreneurial support, social, cultural, political and economic factors. Based on these conflicting results, more research studies in the field of EI within developing countries are needed.

Previous research and definitions of EI divide the construct into two major areas of importance. The first is intention to start a venture (Shapero, 1975; Ajzen, 1991; Boyd and Vozikis, 1994; Thompson, 2009). The second area is attitude, experience and behaviour (Boyd and Vozikis, 1994), which, in the context of entrepreneurial intentionality, indicates that individuals are inclined to EI based on the personal factors, such as ECs, capabilities, personality traits and personal characteristics. In addition to this, EI can be broken down into two categories, namely an intention to create a new venture or an intention to create a new value within an existing business venture (Bird, 1988). Previous scholars such as Zhao et al. (2010) conducted meta-analytical research, which examined the relationship of personality and EI with two different stages in the entrepreneurial process. In their study, EI was drawn from a sample of nascent entrepreneurs within developed countries (Zhao et al., 2010).

### Entrepreneurial Competencies

Entrepreneurial competencies have been defined as the knowledge, skills, abilities, values, attitudes, personality and expertise that lead to entrepreneurial action (Kiggundu, 2002; Morris et al., 2013) and success (Dixon et al., 2005). Research on ECs has focused on education and training that enhance these competencies (for e.g., Cheetham and Chivers, 1996; Wilson et al., 2004; Sánchez, 2011; Kaur and Bains, 2013; Morris et al., 2013), as well as establishing a list of ECs that can lead to entrepreneurial behaviour (Obschonka et al., 2010). In the study by Morris et al. (2013), pre- and post-measures were employed, of which the findings indicated to a substantial enhancement in the ECs, which confirms that competencies can be learnt. Dermol and RoŽman (2014) agree that teaching and supporting ECs are critical as the outcomes can lead to entrepreneurs that are more innovative, creative and can develop and manage ventures. Baron (2008) and Morris et al. (2013) postulate that, although researchers have identified characteristics, values and cognitive approaches related to entrepreneurial success, the competencies that facilitate entrepreneurial action remain elusive. Similarly, the development of these higher-order competence constructs, the progressive role of multiple actors and how their relationships advance in the early stages of venture development are omitted in our understanding of ECs (Rasmussen et al., 2011).

We acknowledge that existing entrepreneurs will view a new venture very differently from someone venturing for the first time, especially with regard to risk taking and making mistakes (Ucbasaran et al., 2010). Hence, as stated previously, ECs should be measured at different stages of the venture lifecycle (Bird, 1995; Baum et al., 2001; Colombo and Grilli, 2005; Mitchelmore and Rowley, 2010). Markman and Baron (2003) suggest that the closer the match between the personal characteristics of an entrepreneur and the requirements of being an entrepreneur, the more successful they will be. For most pre-entrepreneurial ventures, competencies are not freely available but have to be fostered during the early development stages (Rasmussen et al., 2011). In analysing the mediating effect of competence categories such as emotional, social and cognitive competencies, these competencies were found to predict EI within students (Bonesso et al., 2018). By using the three “blocks” of competence categories such as professional competencies, social competencies and personal competencies, the empirical findings suggest that managerial competencies, which include all three categories, are associated with performance in SMEs (Veliu and and Manxhari, 2017). Other authors such as Schneider (2017) suggest that ECs can be operationalised by six first-order constructs, including the functional tasks related to managerial skills, entrepreneurial characteristics of self-efficacy and orientations of competition, risk taking and innovation and the founder and innovator identity. Ryan et al. (2009) added to the empirical literature related to the validity and practical utility of emotional, social and cognitive competencies, and found that these categories are most predictive of performance.

#### A Set of Interrelated ECs

As mentioned earlier, there are many different ECs that are deemed as “crucial” to enhance entrepreneurial action and behaviour. In this paper, we adopt the core ECs as developed and validated by Morris et al. (2013). These aforementioned ECs are mostly included in the work by entrepreneurial scholars specialising in competency development.

According to the human and social capital theories, individuals obtain resources in specific environments (environmental inputs), such as their demographic, social and cultural surroundings (Biraglia and Kadile, 2017:172) and personal networks, which may affect future EIs (behaviour) (Ucbasaran et al., 2007). Human capital theory, measured in the form of work experience, level of education, upbringing by entrepreneurial parents and other life experiences, predicts that individuals who possess higher levels of knowledge, skills and other competencies will achieve higher performance outcomes (Martin et al., 2013). Similarly, entrepreneurs with prior entrepreneurial experience are also more likely to engage in entrepreneurial action learning behaviours and achieve better venture performance (Chen and Pan, 2019). Therefore, the human and social capital theories suggest that specific resources in the form of expertise (i.e., the ECs) are linked to the discovery, evaluation, exploitation and managing of uncertain entrepreneurial opportunities while developing broader social networks that are beneficial to the entrepreneurial process (Shane and Khurana, 2003). The acquisition of specific human capital is expected to result in entrepreneurs being more capable to act on opportunities (Wiklund and Shepherd, 2008) and understand the “true” value of those opportunities (Davidsson and Honig, 2003). Consequently, increased human and social capital (i.e., through the ECs) should enhance an entrepreneur's actual and perceived *self-efficacy* to exploit opportunities (Wiklund and Shepherd, 2008; Dimov, 2010), as well as their attitudes towards *exploiting and assessing opportunities* that may, in turn, predict EI (Schlaegel and Koenig, 2014; Fayolle et al., 2015; Miralles et al., 2016). *Self-efficacy* involves the perceived ability to perform certain behaviours (Liguori et al., 2017) and has been shown to predict EI (Kolvereid, 1996; Bronowitz and Rader, 2008; Wakkee et al., 2010; Pfeifer et al., 2016), as well as moderate the relationship between EI and entrepreneurial action (Boyd and Vozikis, 1994). Through EI, nascent entrepreneurs exhibit higher levels of self-efficacy before they start a business, which results in higher levels of entrepreneurial behaviour for future endeavours (Hsu, 2011; Hsu et al., 2017). Entrepreneurial self-efficacy of college students has also been found to have a significant positive effect on EI and their entrepreneurial attitude, which plays a partial intermediary role in this relationship (Liu et al., 2019). Gielnik et al. (2018) found in their study that *opportunity recognition* could positively and significantly predicted EI. Similarly, EI is seen as the mental force that assesses and realises the value of a new business opportunity (Cha and Bae, 2010). With no intention, opportunities cannot be assessed and without the ability to *assess opportunities*, EI and opportunity are unlikely to be realised (Morrison et al., 2003).

As explained above, this interrelatedness of ECs is also likely the case for other ECs such as *opportunity recognition and assessment* as both involve the perception of the opportunity (McMullen and Shepherd, 2006). *Opportunity assessment and recognition* only differs with respect to the level of involvement required in the assessment of an opportunity and the locus of assessment (i.e., the assessment of a first-person opportunity as opposed to a third-person opportunity) (McMullen and Shepherd, 2006; Schlaegel and Koenig, 2014). It is, therefore, contended that individual ECs covary between one another. ECs may bring forth reputational value for entrepreneurs, which can aid in entrepreneurial action (Gielnik et al., 2018) by relying on this reputation to *convey their vision* in a more compelling way. This should enhance the actual and perceived *self-efficacy of* an entrepreneur to exploit opportunities (Dimov, 2010) and, in turn, more strongly predict EI (Schlaegel and Koenig, 2014; Miralles et al., 2016). A recent study by Biraglia and Kadile (2017) found a strong positive relationship between self-assessed *creativity* and EI, which was mediated through entrepreneurial self-efficacy. Hu et al. (2018) found further evidence for the argument that entrepreneurial alertness has a fully mediation effect on the relationship between creativity, proactive personality and EI. Because domain-relevant knowledge acquired through prior experience is a highly relevant component of creativity (Amabile, 1983; Baer, 2012), entrepreneurs should be able to draw on this prior entrepreneurial experience in the form of domain-specific knowledge that can enhance creativity levels and, consequently, EI (Biraglia and Kadile, 2017). Morris et al. (2013) describe *value creation* as the “capabilities of developing new products, services and/or business models that generate revenues exceeding their costs and produce sufficient user benefits to bring about a fair return.” Scholars such as Gorman et al. (1997), Feldman and Bolino (2000) and Sternberg (2004) have suggested that innovative individuals are motivated to become self-employed. Because innovation involves the implementation of creative ideas or solutions (Perry-Smith and Mannucci, 2017), entrepreneurs will be better able to act on this innovative intentionality (Wiklund and Shepherd, 2008), leading to stronger predictive power of EI. *Building and maintaining diverse social networks* plays an essential role in developing EI among entrepreneurs (Kefela, 2011; Zafar et al., 2012). Entrepreneurs through several environmental inputs are thus expected to have broad social networks and improved effectiveness in developing network ties (Mosey and Wright, 2007). Similarly, nascent entrepreneurs have likely accumulated more social capital (Ucbasaran et al., 2007), which is a salient factor in developing EI among entrepreneurs (Zafar et al., 2012).

From the discussion above, it is evident that ECs are interactional constructs, meaning they are dependent on, and interact with, individual personalities, the environment and behaviour as defined by the situational environment (Mitchelmore and Rowley, 2010:95). Accordingly, the ECs are expected to interact and covary with one another, as recently supported in the study by RezaeiZadeh et al. (2017). For e.g., ECs related to *creative problem solving* appear to significantly enhance other ECs such as *networking* and leadership (RezaeiZadeh et al., 2017). Therefore, we adopt the view in this paper that ECs can more effectively be developed and learnt by nascent entrepreneurs if these ECs are categorised in higher-order competence constructs, which are discussed in the next subsection.

#### Categorising ECs Into Higher-Order Competence Constructs

As indicated in A Set of interrelated ECs section, many scholars found that competencies can be categorised and project a better outcome than competencies that are developed in isolation (Lado et al., 1992; Eden and Ackermann, 2000; Harmsen et al., 2000; Patterson et al., 2002). In many cases, a specific combination of competencies or categorising competencies into higher-order constructs are known to achieve a greater success (Harmsen et al., 2000). Patterson et al. (2002) found that a set of six self-directed learning competencies are not mutually exclusive but are interrelated in such a way that by using all or a combination of them simultaneously directs and controls students' learning. Scholars such as Eden and Ackermann (2000) and Lado et al. (1992) use the term “distinctive competencies.” Distinctive competencies is defined by Eden and Ackermann (2000) as “those particular strengths within an organisation that are very difficult to emulate,” and are the features of an organisation that underpin long-term success. Distinctive competence is often the combination of a particular pattern of interrelated competencies, where it is the pattern that is distinctive (Eden and Ackermann, 2000). In their research, Lado et al. (1992) examine sustainable competitive advantage linking the four components of a firm's “distinctive competencies” (managerial competencies and strategic focus, resource-, transformation- and output-based competencies) that are synergistically related. The results by Harmsen et al. (2000) support a nonfunctional and broad perspective of how bundles of competencies interact and impact on the success and establish a positive overall contribution to product development. About 10 competencies (areas of importance) were identified for achieving company objectives. On account of investigating the competencies needed by individual engineers, competencies are understood to be interrelated rather than separate within the Definition and Selection of Competencies (DeSeCo) framework (Male et al., 2011). In the field of entrepreneurship, it has been found that EI influences the attitude, skills and behaviour of pre-entrepreneurs and nascent entrepreneurs (Fayolle et al., 2015; Hsu et al., 2017). Similarly, existing entrepreneurs, should however, have some or most of the ECs as they have already proceeded through several stages of the business life cycle. However, this is not the case for nascent entrepreneurs, and a combination of ECs need to be developed.

**Figure 1:**
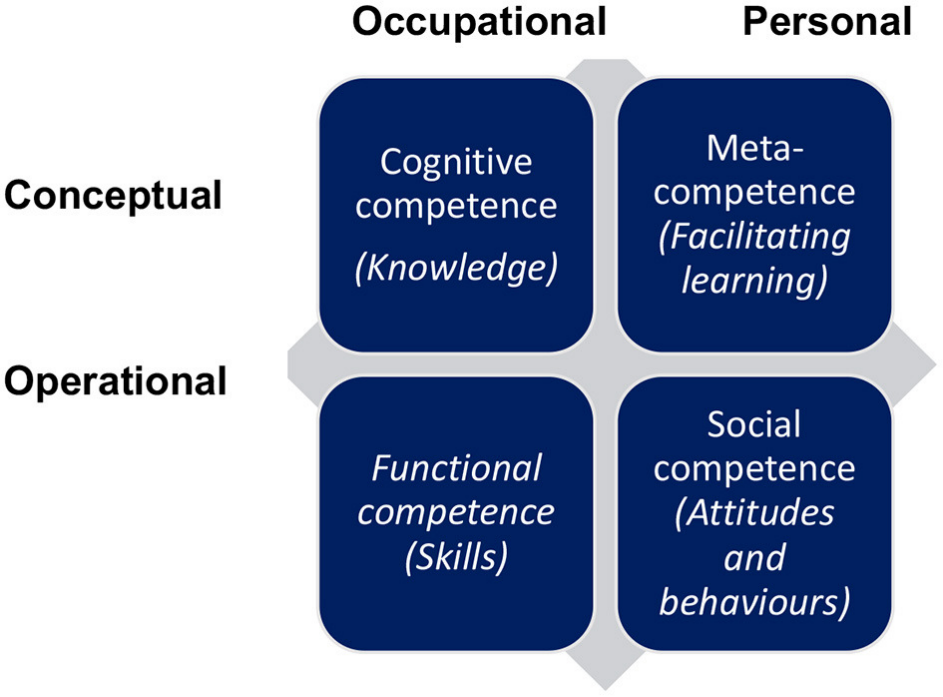
A unified typology of competencies. *Source*: Adapted from Winterton et al. (2006).

Winterton et al. (2006) developed a typology for categorising ECs. We adopt this approach by grouping ECs in the study of Morris et al. (2013) into four categories. A unified typology of competence, knowledge and skills that are necessary for particular occupations, as developed by Winterton et al. (2006) is illustrated in Figure 1. Skills are captured by functional competence, knowledge is captured by cognitive competence, attitudes and behaviours are captured by social competence while meta-competence, which is rather different from the first three dimensions, is concerned with facilitating the acquisition of the other substantive competence (Winterton et al., 2006:41). Fayolle et al. (2015) tested the relationship between EI and cognitive and behavioural competencies, not necessarily focusing on individual ECs, and found strong relationships between EI and cognitive and behavioural competencies.

**Figure 2:**
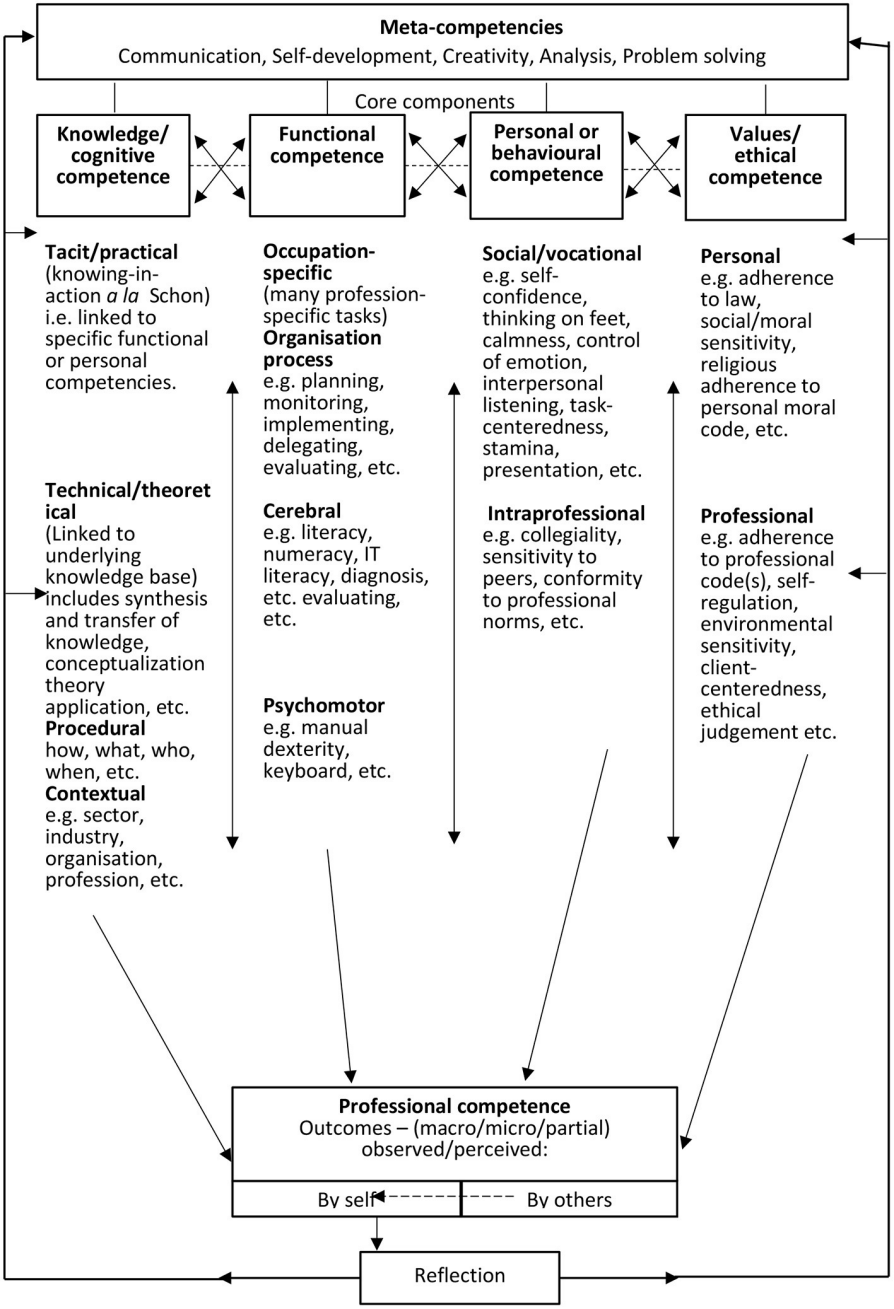
The categorisation of meta-competencies. *Source*: Adapted from Cheetham and Chivers (1996:27).

Furthermore, Cheetham and Chivers (1996) also developed and tested meta-competencies, as illustrated in Figure 2, and divided it into four categories very similar to these of Winterton et al. (2006).

For the purpose of this paper, we therefore adopt the four categories by Winterton et al. (2006) and Cheetham and Chivers (1996), namely (1) cognitive competencies; (2) social/personal competencies; (3) functional competencies and (4) meta-competencies. These authors define each of these higher-order categories as follows:

- *Cognitive competence* refers to underpinning theory and concepts as well as informal tacit knowledge gained experientially; knowledge, the “know that” is underpinned by understanding, the “know why.”
- *Functional competence* refers to skills or know-how and things that a person should be able to do and to demonstrate.
- *Social/personal competence* refers to behavioural competencies or knowing how to behave; some behaviours and attitudes related to EC are having a positive attitude towards change and showing initiative.
- *Meta-competence* refers to as a comprehensive concept of the multidimensional construction of competence; it further refers to the element that facilitates the acquisition of the other competencies.

Based on the definitions and discussion in A Set of interrelated ECs section surrounding each individual EC as presented by Morris et al. (2013), the ECs in this paper are categorised and presented in Table 1.

**Table 1:** Grouping entrepreneurial competencies (ECs) into higher-order competence categories.

**Cognitive**	**Social/personal**	**Functional**	**Meta**
•Conveying a compelling vision	•Building and using networks	•Value creation through innovation	
•Creative problem solving	•Self-efficacy		None
•Opportunity recognition	•Tenacity/perseverance		
•Opportunity assessment			

### The EI-Competency Relationship for Nascent Entrepreneurs

**Figure 3:**
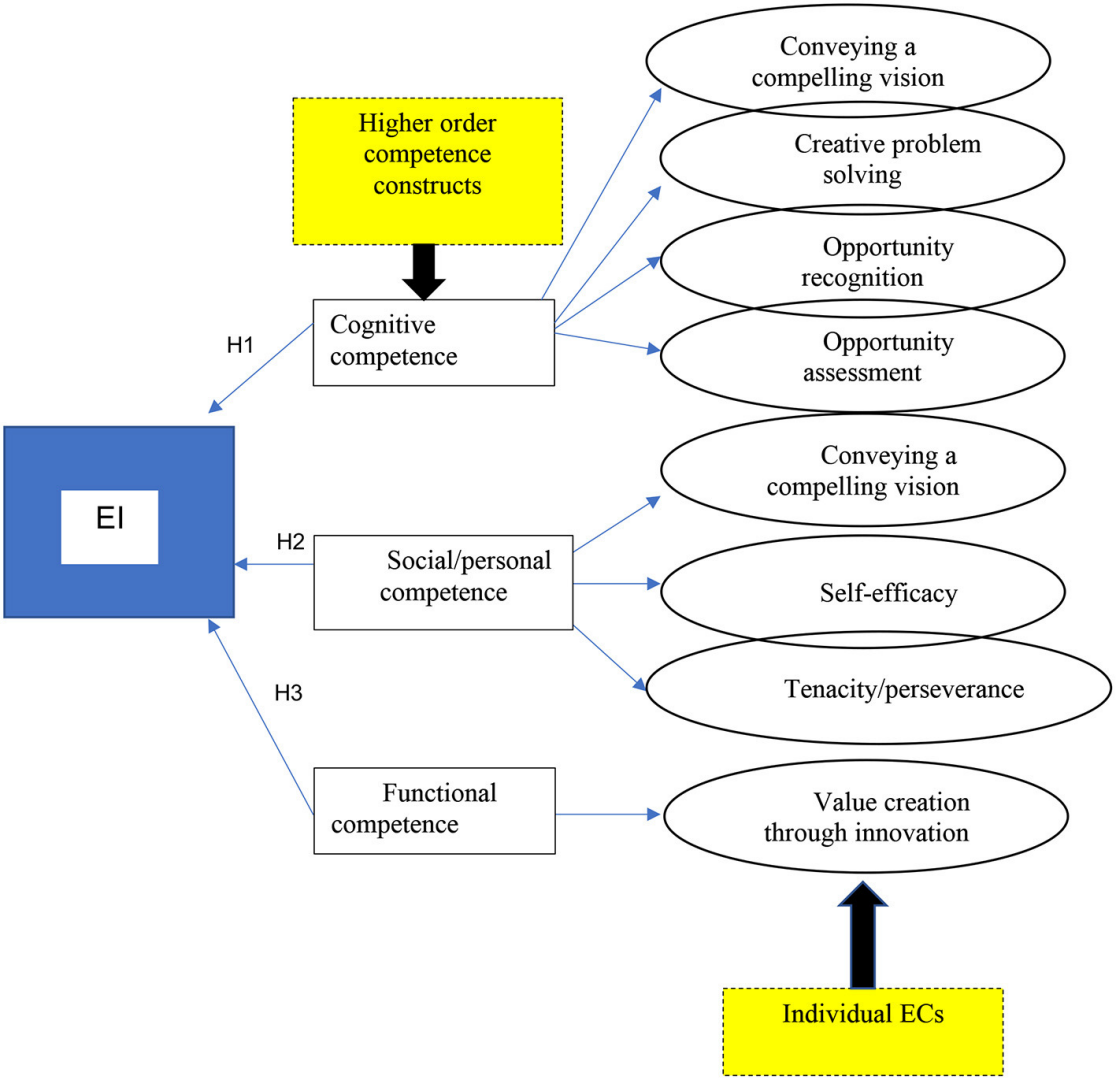
The entrepreneurial intention- (EI-) competency higher-order category model.

In turn, TPB implies that intentions are determined by attitudes, and attitudes are affected by “exogenous effects” such as competencies, traits and situational variables (Krueger, 1993). Some scholars such as Hazlina Ahmad et al. (2010) argue that personality is condensed in ECs and we therefore acknowledge that personality traits are embedded in ECs. Furthermore, there is a discrepancy in the literature relating to the different personality traits and ECs and they are often used interchangeably. Hmieleski and Corbett (2006) argue that personality, cognition, motivation and improvisation are the good predictors of EI. A few scholars such as Obschonka et al. (2010) observe that: “Entrepreneurship research to date has rarely addressed early antecedents of entrepreneurial activities, such as early ECs. As expected, entrepreneurial personality, early ECs and intention were associated.” SCT provides a useful model (Bandura, 1986) for understanding human action and its consequences (Hmieleski and Baron, 2009). In the formulation of SCT, Bandura proposes that learning, motivational and behavioural actions are the outcomes of a complementary interaction between the three distinct aspects, namely (1) environmental input; (2) personal factors and (3) behavioural outcomes (Bandura, 1989, 2006; Biraglia and Kadile, 2017). Personal factors include physiological features, suppositions, perceptions, affect and cognitive capabilities (Biraglia and Kadile, 2017). Therefore, the view is supported that higher-order constructs, such as cognitive and personal (social) competencies, are related to EI. Based on the preceding literature review, an overarching theoretical framework is provided in Figure 3 and will be tested in the analyses.

As depicted in Figure 3, the higher-order competency constructs are presented as cognitive, social/personal and functional competence. Each of the individual ECs that are related to each of the higher-order competency constructs is illustrated. From this discussion and Figure 2, as meta-competence is associated with facilitating the acquisition of the other substantive competence, we do not view the individual ECs that are specifically categorised under the construct of meta-competence. Therefore, the hypothesised statements are introduced for the cognitive, social/personal and functional competences only:

*H1*: Cognitive competence, consisting of conveying a compelling vision; creative problem solving; opportunity recognition and opportunity assessment, is positively related to EI for nascent entrepreneurs.
*H2*: Social/personal competence, consisting of building and using networks; self-efficacy and tenacity/perseverance, is positively related to EI for nascent entrepreneurs.
*H3*: Functional competence, consisting of value creation through innovation, is positively related to EI for nascent entrepreneurs.

### Method and Sample

To address the research hypotheses, a quantitative research approach was followed, employing a structured research questionnaire (survey). The study used a nonprobability sampling procedure, specifically judgemental/purposive sampling. With purposive sampling, the sample is arbitrary and subjectively selected (Cooper and Schindler, 2011) to fulfil a purpose of providing answers to the study's research questions and objectives. The target population was nascent entrepreneurs. South African organisations, such as the University of Pretoria (tertiary institution), National Youth Development Agency (governmental organisation) and the Gauteng Enterprise Propeller (nongovernmental organisation), were used to source the contact details of nascent entrepreneurs. The survey was emailed to approximately 1,450 respondents, and 330 hard copies were distributed. The final realised sample was 203, which represented a low response rate of 8.8%. Although the sample was selected arbitrary, the sample elements were selected based on their adherence to the determinants mentioned in the next section.

### Categorising Nascent Entrepreneurs

The operational definition of nascent entrepreneurs in the GEM report (Herrington et al., 2019) was taken into consideration as it defines nascent entrepreneurs as those individuals who are in the process of setting up a business (Herrington et al., 2019). They are therefore individuals who are identified as taking steps to start a new business through a perceived opportunity or by a personal aspiration but who had not yet succeeded in making the transition to new business ownership (Lichtenstein et al., 2007). Scholars interested in nascent entrepreneurs tend to focus their attention not on a single act of opportunity exploitation, but more on the series of actions in new venture emergence (Delmar and Shane, 2004; Lichtenstein et al., 2007; Kim et al., 2015). For example, nascent entrepreneurs often look for and purchase facilities and equipment, seek and obtain funding, organise teams and dedicate their time and energy to their business (Carter et al., 1996). For the purpose of this paper, an individual was regarded a nascent entrepreneur if s/he had “initiated at least one gestation activity for a current, an independent start-up by the time of the measurement.” Gestation activities refer to actively commencing the process of establishing a business (Davidsson and Honig, 2003) including having prepared a business plan; developed an idea; recognised an opportunity; developed a new product or service; tested the product or service on customers; conducted market research; applied for a patent or trademark; applied for financial assistance; started with marketing activities; saved money to start a business; undergone any entrepreneurial education or training with the goal of starting a business and gathered other resources to start a business.

### Measures

The 10-item Individual Entrepreneurial Intention Scale (IEIS), developed by Thompson (2009), was used in this paper to measure EI for nascent entrepreneurs. The ordinal scale used was from 1 (very untrue) to 4 (very true). In Thompson's study, the scale's Cronbach's alpha coefficient of internal consistency proved to be 0.89 for 450 randomly selected convenience respondents; 0.84 for 160 student respondents and 0.86 for an international sample of 947. Hence, the scale seemed to have acceptable internal consistency. Confirmatory factor analysis (CFA) was conducted to determine whether the data fit this EI scale.

To identify the relevant EC scale, Morris et al. (2013) implemented a multi-round Delphi technique to generate an essential list of 13 core ECs. The survey was adapted in terms of language and the number of items per EC to fit within the developing country context, therefore an exploratory factor analysis (EFA) was considered and not a CFA. EFA was employed for each of the competencies, using principal axis factoring extraction and promax rotation, to determine the unidimensionality of each of the constructs. Convergent validity was confirmed by means of composite reliability (CR) for the constructs in the study under The validity and reliability of the scales Section and presented in Table 2.

**Table 2:** Composite reliability (CR) scores.

**Individual ECs**	**AVE (Average)**	**CR**
Conveying a compelling vision	0.462	0.719
Building and using networks (Maintain contacts)	0.626	0.869
Value creation through innovation (Observing customer usage)	0.443	0.704
Self-efficacy	0.629	0.834
Building and using networks (Participate in community events)	0.530	0.693
Creative problem-solving/ imaginativeness	0.501	0.751
Tenacity/perseverance	0.479	0.733
Opportunity recognition	0.502	0.665
Opportunity assessment	0.466	0.635
Value creation through innovation (Challenge status quo)	0.548	0.702
Entrepreneurial intention (EI)	0.21	0.614

The Likert-type response scale used was from 1 (strongly disagree) to 5 (strongly agree) and an AVE of six items was measured per EC. In the study by Morris et al. (2013), the Cronbach's alpha coefficient was tested individually for each of the ECs and varied between 0.62 as the lowest and 0.97 as the highest. Therefore, the EC scale also seemed to have acceptable internal consistency. Both the EI as well as the EC scales were administered in English.

The use of different scales (very untrue to very true) for EI and the five-point agreement scale for the ECs, as well as the reversed score items in the instruments, alleviate potential common method bias.

### Data Analysis

In order to assess normality, individual item statistics revealed that all items, except items 86–88 and 93 had skewness and the kurtosis values between −2 and +2, therefore confirming the assumption of univariate normality (George and Mallery, 2010) for these items. For items 86–88, the skewness value was within the range of −2 to +2 with the kurtosis values of 2.79, 3.97 and 2.54, respectively. Ryu (2011) found that skewness = 2 and kurtosis = 7 appeared to be the level of violation of multivariate normality at which the influence of the violation became severe when conducting SEM. Only item 93 had values above 2 and 7. However, as it was only an item that violates this assumption, ML estimation used in SEM was deemed appropriate.

Correlations between the individual variables were employed to determine the strength and direction of the individual relationships and to detect multicollinearity. Model estimation and specification used SEM to conduct structural analysis and these analyses were performed by using SPSS (version 24) and AMOS (version 24). The structural analysis determine whether a relationship existed between the latent variables and robust SEs were estimated by means of bootstrapping (refer to **Table 5** for bootstrapping of the parameters).

### The Validity and Reliability of the Scales

Confirmatory factor analysis was conducted and indicated a non-acceptable fit for EI [root mean square error approximation (RMSEA) = 0.096; incremental fit index (IFI) = 0.750, comparative fit index (CFI) = 0.744 and minimum discrepancy per degree of freedom = 4,172]. A possible reason why CFA indicated a non-acceptable fit was due to a wide range of perception ratings of the items by the respondents (the mean values range between 2.5 and 3.68 with SDs ranging between 0.585 and 0.977), therefore EFA is undertaken to determine the potential subdimensions of EI.

Subsequently, EFA was conducted to determine the factor structure for both EI and each of the ECs. Cronbach alpha coefficients of more than 0.70 are typically regarded as acceptable (Nunnally, 1978) when using existing instruments. However, 0.6 is regarded acceptable in exploratory research (Bagozzi and Yi, 1988; Hair et al., 2010). In a study by Farrington et al. (2012), low Cronbach alpha values of between 0.50 and 0.60 were considered sufficient and useful to the study, which measured entrepreneurial attributes in three different countries, including South Africa (Antonites and Nonyane-Mathebula, 2012).

As the Cronbach alpha value is known to be influenced by the number of items in a scale and is a lower bound estimate of reliability, reliability was also assessed through the CR score in Table 2. These values ranged between 0.614 and 0.869, and consider acceptable as they were all more than 0.6 (Hair et al., 2010). The AVE variance extracted should be higher than the minimum threshold of 0.5. However, according to Fornell and Larcker (1981), even if AVE is less than 0.5, but CR is higher than 0.6, the convergent validity of the construct is still adequate, therefore all the constructs in the study are deemed acceptable for analysis in the SEM.

#### Factor Analysis: ECs

As EFA was conducted per EC scale, unidimensionality was indicated for 10 of the 12 ECs; and 2 of them, value creation, and building and using networks, resulted in two sub-factors each. The reliability analysis conducted on the constructs retained for further analysis is displayed in Table 3. A total of four factors were eliminated as they failed to contribute to a simple factor structure and satisfy the minimum criteria of a primary factor loading of 0.4 or above, and/or no cross-loading exceeding 0.3. These factors and their Cronbach's values were risk management/mitigation (0.34), resource leveraging (0.44), maintain focus yet adapt (0.46) and resilience (0.41). No substantial increases in alpha for any of the scales could have been achieved by eliminating more items. Based on the analysis, 10 competencies (i.e., distinct factors) were indicated as internally consistent, resulting in 8 factors and 4 sub-factors being included for further testing. These factors, the Cronbach's alpha values, and composite scores for each factor are calculated by using the means and SD of the variables included in each factor and are presented in Table 3.

**Table 3:** Descriptive statistics and correlations between the composite scores.

**Factors**	**Mean**	**SD**	**Opportunity** **recognition**	**Opportunity assessment**	**Conveying a compelling vision**	**Tenacity/** **perseverance**	**Creative problem-solving/ Imaginativeness**	**Value creation through innovation (Challenge status quo)**	**Value creation through innovation (Observing customer usage)**	**Self-efficacy**	**Building and using networks (Maintain contacts)**	**Building and using networks (Participate in community events)**
**Pearson's correlations between composite scores**												
Opportunity recognition	4.0567	0.71590	(1)	0.462^**^	0.481^**^	0.476^**^	0.625^**^	0.422^**^	0.492^**^	−0.173^*^	0.306^**^	0.144
Opportunity assessment	4.2591	0.78245	0.462^**^	(1)	0.506^**^	0.524^**^	0.541^**^	0.410^**^	0.472^**^	−0.292^**^	0.349^**^	0.176^*^
Conveying a compelling vision	4.5228	0.58725	0.481^**^	0.506^**^	(1)	0.457^**^	0.564^**^	0.379^**^	0.439^**^	−0.221^**^	0.354^**^	0.124
Tenacity/perseverance	4.1849	0.70350	0.467^**^	0.524^**^	0.457^**^	(1)	0.564^**^	0.416^**^	0.538^**^	−0.352^**^	0.234^**^	0.124
Creative problem-solving/Imaginativeness	4.3205	0.67738	0.625^**^	0.541^**^	0.564^**^	0.564^**^	(1)	0.465^**^	0.668^**^	−0.383^**^	0.341^**^	0.175^*^
Value creation through innovation (Challenge status quo)	4.3333	0.55520	0.422^**^	0.410^**^	0.379^**^	0.416^**^	0.465^**^	(1)	0.537^**^	−0.166^*^	0.272^**^	0.135
Value creation through innovation (Observing customer usage)	4.0374	0.67894	0.492^**^	0.472^**^	0.439^**^	0.538^**^	0.668^**^	0.537^**^	(1)	−0.244^**^	0.263^**^	0.182^*^
Self-efficacy	3.1301	1.26115	−0.108	−0.292^**^	−0.221^*^	−0.352^**^	−0.383^**^	−0.166^*^	–.244^**^	(1)	−0.247^**^	−0.096
Building and using networks (Maintain contacts)	3.2660	1.11012	0.144^**^	0.349^**^	0.354^**^	0.234^**^	0.341^**^	0.272^**^	0.263^**^	−0.247^**^	(1)	0.532^**^
Building and using networks (Participate in community events)	2.9801	1.10756	−0.173^*^	0.176^*^	0.124	0.124	0.175^**^	0.135	0.182^*^	−0.096	0.532^**^	(1)

** *Correlation is significant at the 0.01 level (two-tailed)*.

* *Correlation is significant at the 0.05 level (two-tailed)*.

*Cronbach's alpha values are given in brackets*.

According to Kline (2011), the minimum sample sizes for both precision and power varied widely across the different models and extent of missing data. Minimum sample sizes for factor analysis models normally ranges from 30 to 460 cases, depending on the number of factors (1–3), the number of indicators per factor (3–8), the AVE correlation between indicators and factors (0.50–0.80), the magnitude of factor correlations (0.30–0.50) and the extent of missing data (2–20% per indicator).

#### Correlations

The Pearson's correlation coefficient was employed to determine the strength and direction of the relationship between each pair of ECs. Table 3 shows that the relationship between all ECs was positive, except for the relationships between self-efficacy and each of the other ECs. The values ranged between 0.124 (weak correlation) and 0.668 (between value creation through innovation (sub-factor: observing customer usage and creative problem-solving). These correlation levels indicated strong evidence of no multicollinearity and discriminant validity between the set of ECs.

## Findings

The total sample consisted of 203 nascent entrepreneurs of which 33% were women and 67% were men. The AVE age was 30 with the youngest respondent aged 18 and the oldest aged 71 years. Of the respondents, 47.8% had completed a secondary school level education, with 17.7% having obtained a tertiary qualification (University or Technikon degree). The majority of the nascent entrepreneur respondents indicated that they are going to operate their businesses in the services, agriculture and manufacturing industries.

### Hypothesis Testing

**Figure 4:**
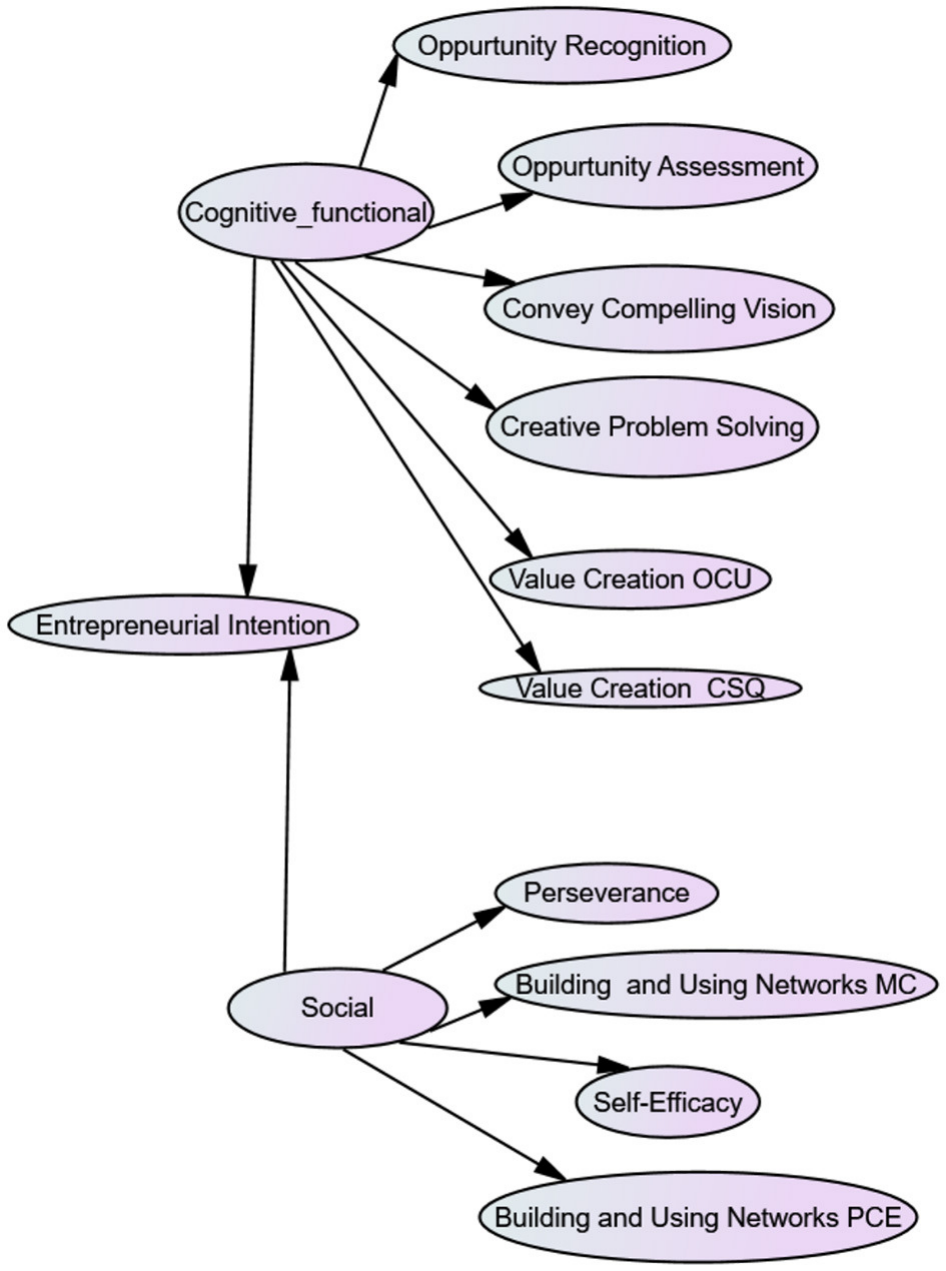
Model representation of the tested EI-competency higher-order category model.

Structural equation modelling was conducted to test the three hypotheses that are graphically illustrated in Figure 3. The relationship between EI and each higher-order category (cognitive competence in H1; social/personal competence in H2 and functional competence in H3) with their representation of the individual ECs is tested. This model indicated an almost acceptable model fit with the following goodness-of-fit measures (CFI−0.844; IFI−0.847; TLI−0.826 and RMSEA−0.069). For the goodness-of-fit measures, CFI, IFI, TLI values above 0.9 and RMSEA values between 0.08 and 0.05 indicate a reasonably well-fitting model while a RMSEA value below 0.03 represents excellent fit (Hooper et al., 2008). However, due to multicollinearity between the cognitive and functional category (0.921), the decision was taken to combine the cognitive and functional higher-order competency constructs. Therefore, the combined cognitive and functional as well as social/personal competence resulted in two higher-order competence constructs that were tested with their individual ECs in relation to EI. The new model representation is illustrated in Figure 4.

From Table 4, it is evident that this model had CFI (0.900) and IFI (0.902) values above 0.9 with a TLI (0.886) value very close to 0.9. Overall, the other goodness-of-fit measures indicated a reasonably well-fitting model (RMSEA, 0.056).

**Table 4:** Measures of the model fit of the structural equation modelling (SEM) for the cognitive and functional as well as the social/personal categories (nascent sample).

**Model fit indicators**	**Model (*n* = 203)**
χ^2^/*(Df)*	1.635
Tucker-Lewis index (TLI)	0.886
Incremental fit index (IFI)	0.902
Comparative fit index (CFI)	0.900
Root mean square error approximation (RMSEA)	0.056

Because this SEM model with the combined cognitive and functional higher-order competence constructs provided an acceptable model fit, the strength of the relationships (correlations) between the variables and the associated significance levels for this model was analysed, as shown in Table 5. Strength thresholds (0–0.2 = weak; 0.2–0.4 = mild/modest; 0.4–0.6 = moderate; 0.6–0.8 = moderately strong and 0.8–1.0 = strong) are used in accordance with Pallant (2001). Opportunity recognition (0.922); opportunity assessment (0.909); conveying a compelling vision (0.828) and creative problem-solving (0.946) have a positively strong relationship with the cognitive and functional higher-order competence. Both the value creation ECs (0.735 and 0.780, respectively) have a positively moderately strong relationship with the cognitive and functional higher-order competence. Perseverance (0.253) and self-efficacy (−0.361) have a modest relationship with social/personal higher-order competence. However, this relationship is negative for self-efficacy and social/personal competence. Both the building and using networks ECs (0.831 and 0.963, respectively) have a positively strong relationship with the social/personal higher-order competence. When testing the strength of the relationships between EI and the cognitive and functional higher-order competence, a positively strong relationship was found. On the other hand, a negatively weak relationship was found between EI and the social/personal higher-order competence. A significant positive relationship is evident between all of the variables except between perseverance and social/personal competence and EI and social/personal competence. The positive relationships are in agreement with previous scholars (Kolvereid, 1996; Bronowitz and Rader, 2008; Wakkee et al., 2010; Pfeifer et al., 2016) who found a positively strong relationship between most of the ECs in the cognitive and functional competence category and EI. Furthermore, as indicated, robust SEs were estimated by means of bootstrapping (refer to Table 5) and all of the bootstrapped SE seem to be consistent.

**Table 5:** Bootstrapped standardised regression weights and SE.

**Relationship variables**	**Standardised regression weights**	**SE (Bootstrapping)**
Opportunity recognition←Cognitive and Functional competence	0.922^***^	0.059
Opportunity assessment←Cognitive and Functional competence	0.909^***^	0.050
Conveying a compelling vision←Cognitive and Functional competence	0.828^***^	0.064
Creative problem-solving←Cognitive and Functional competence	0.946^***^	0.038
Value creation through innovation (Challenge status quo)←Cognitive and Functional competence	0.735^***^	0.077
Value creation through innovation (Observing customer usage)←Cognitive and Functional competence	0.780^***^	0.053
Perseverance←Social/personal competence	0.253	0.097
Self-efficacy←Social/personal competence	−0.361^*^	0.105
Building and using networks (Maintain contacts)←Social/personal competence	0.831^**^	0.054
Building and using networks (Participate in community events)←Social/personal competence	0.963^**^	0.068
EI←Cognitive and Functional competence	0.817^***^	0.236
EI←Social/personal competence	−0.089	0.176

*EI, Entrepreneurial intention*.

* *p < 0.05*,

** *p < 0.01*,

*** *p < 0.001*.

## Discussion of the Findings

The EI–EC relationship was tested in a SEM model on a sample of nascent entrepreneurs to explore the higher-order competence constructs and their individual ECs in relation to EI. Based on the literature review, Figure 3 graphically illustrated the four higher-order competence constructs and the individual ECs that are categorised under each higher-order construct. However, this model did not provide an acceptable model fit and due to multicollinearity between the cognitive and functional category (0.921), SEM was rerun by combining the cognitive and functional higher-order constructs whereby an acceptable model fit was found. In the SCT framework, ECs are seen as personal factors (Biraglia and Kadile, 2017) rather than a part of behaviour. Competencies are only observable if actual actions are taken by individuals in particular situations. Individual capacities or dispositions, external demands or dispositions and contexts are all part of the complex nature of competencies (OECDE, 2005). This finding aligns with, and furthers the understanding of, the findings of RezaeiZadeh et al. (2017) that ECs are interdependent and can be categorised together. Hence, support is found for the suggestions by Mitchelmore and Rowley (2010) that ECs are interactional constructs that are dependent on, and interact with, the personality (including the other ECs) of an individual and can be categorised into higher-order constructs. We find support for the first and third hypotheses, H1: cognitive competence, consisting of conveying a compelling vision; creative problem-solving; opportunity recognition and opportunity assessment, is related to EI for nascent entrepreneurs. H3: functional competence, consisting of value creation through innovation, is related to EI for nascent entrepreneurs, is combined with cognitive competence in our SEM model. Hence, we can confirm our assumption in the literature review where TPB implies that intentions are determined by attitudes, and attitudes are affected by competencies (Krueger, 1993), we can confirm that higher-order competence constructs with individual ECs categorised under the cognitive and functional constructs can predict EI.

There is no evidence supporting the second hypothesis (H2) stating that social/personal competence, consisting of building and using networks; self-efficacy and tenacity/perseverance, is related to EI for nascent entrepreneurs. Our findings suggest that EI has a negative and weak relationship with the social/personal higher-order construct, and perseverance and self-efficacy cannot be categorised in the higher-order competence construct of social/personal competence. This might be due to the fact that self-efficacy should perhaps be investigated as a higher-order competence construct on its own as the previous literature studies indicated such a strong relationship with EI (Kolvereid, 1996; Bronowitz and Rader, 2008; Wakkee et al., 2010; Pfeifer et al., 2016).

We also tested the strength and direction of these relationships for the acceptable model. The strongest positive relationships were observed between EI and cognitive and functional higher-order competence, specifically creative problem-solving, conveying a compelling vision, opportunity recognition and opportunity assessment with this higher-order construct. Our findings agree with previous scholars that EI is closely linked to opportunity recognition, opportunity assessment (Shaver and Scott, 1991; Krueger et al., 2000) and value creation (Gorman et al., 1997; Feldman and Bolino, 2000; Sternberg, 2004). The weakest relationship is observed between EI and the social/personal higher-order competence, specifically self-efficacy indicated a negative relationship with social/personal competence. The literature on self-efficacy indicated a positive relationship with EI (Kolvereid, 1996; Bronowitz and Rader, 2008; Wakkee et al., 2010; Pfeifer et al., 2016), and specifically nascent entrepreneurs exhibit higher levels of self-efficacy before they start a business, which results in higher levels of entrepreneurial behaviour for future endeavours (Hsu, 2011; Hsu et al., 2017). However, in this paper we cannot confirm this when self-efficacy is categorised into a higher-order competence construct of social/personal competence. Future models could test self-efficacy as a higher-order construct with certain individual ECs such as opportunity recognition (Wiklund and Shepherd, 2008; Dimov, 2010), as well as opportunity assessment (Schlaegel and Koenig, 2014; Fayolle et al., 2015; Miralles et al., 2016) categorised under self-efficacy as these ECs have strong relationships with self-efficacy. We acknowledge that higher-order constructs in EC research requires further testing and investigation to explore possible best practise models for teaching these ECs.

## Conclusion

As emphasised in the literature, individuals with high levels of EI does not necessarily start businesses or engage in entrepreneurial behaviour. The missing link between EI and action could be the lack of having adequate ECs. There has been little in-depth research on ECs and their complex relationship with EI (i.e., Boyd and Vozikis, 1994; Hayton et al., 2002; Thompson, 2009; Turker and Sonmez Selçuk, 2009; Prodan and Drnovsek, 2010; Arasti et al., 2012; García-Rodríguez et al., 2015). We used several theories as an underlying framework to explore the EI–EC relationship. The SCT (Bandura, 1986) provides a supporting model suggesting a relationship between EI and higher-order constructs under which individual ECs could be categorised. Winterton et al. (2006) and Cheetham and Chivers (1996) introduced four higher-order categories namely: (1) cognitive competencies; (2) social/personal competencies; (3) functional competencies and (4) meta-competencies. However, the relationship of these four higher-order competence constructs with EI could not be supported in this paper. Specifically, model fit was evident when cognitive and functional competence are combined into one higher-order construct. This combined higher-order competence construct and social/personal competence were included in the SEM model testing the relationship with EI. Therefore, we advance theory regarding the relationships between EI and ECs and the higher-order categories for ECs (Mitchelmore and Rowley, 2010). As there is a lack of research measuring the relationship between EI and ECs, specifically in a developing country context on samples other than students, this paper fills that gap. The SCT (Bandura, 1986), social (Coleman, 1988) and human capital theories (Becker, 1993) support the relationship between EI and ECs and also the strength of the model fit when examining nascent entrepreneurs.

We found support in the literature for findings that EI has a positively strong relationship with the cognitive and functional higher-order construct and specifically for opportunity assessment (Morrison et al., 2003); conveying a compelling vision (Cha and Bae, 2010); creative problem-solving (McMullen and Shepherd, 2006) and opportunity recognition (Gielnik et al., 2018). Consequently, entrepreneurship education programmes and educators should be aware that the necessary ECs within the cognitive and functional higher-order construct should be developed and trained simultaneously to enhance EI if we want to encourage more nascent entrepreneurs to make the leap towards owning a business venture. Previous literature studies indicate that self-efficacy is a strong positive predictor of EI (Kolvereid, 1996; Bronowitz and Rader, 2008; Wakkee et al., 2010; Pfeifer et al., 2016). However, when tested as a second-order construct under the social/personal higher-order competence construct, a positive relationship with EI could not be confirmed. By deconstructing this relationship, we recommend that future EI–EC relationships should test self-efficacy as a higher-order competence construct to ensure that nascent entrepreneurs has the best possible “entrepreneurial training package” to enhance the outcome of entrepreneurial behaviour. We further recommend that individual ECs should be developed in conjunction with the higher-order competence construct, specifically the cognitive and functional competence, rather than individually, to increase EI.

The contributions of this paper are three-fold. First, from a theoretical viewpoint, exploring the relationships between EI and higher-order competence constructs have merits as it has received scant research attention to date. We contribute to the SCT, TPB, human and social capital theories by applying the theories in an entrepreneurial context and confirming that there is a relationship between behavioural (EI) and personal (ECs) as well as other higher-order competence constructs. We also contribute to the EI literature by testing and confirming the acceptable model fit in relationship with two higher-order competence constructs. Secondly, from a practical viewpoint, the findings may guide policy interventionists to focus on the right set of ECs and the development thereof in relation to the higher-order constructs identified. These two higher-order constructs, the individual ECs under each and their intricate relationship with EI, provide promising avenues for enhancing entrepreneurial action and development, especially for the cognitive and functional higher-order competence construct. A third contribution of this paper lies in the geographical sphere. Because this research has been conducted in South Africa, we have answered the call for entrepreneurship research from an African perspective (George et al., 2016). The fresh insights into EI and its relationship with competencies have been garnered from a research context distinct from where the constructs themselves originated (Zoogah et al., 2015; George et al., 2016). Established scholars in entrepreneurship research, with a reference to intention and competencies, have explored these constructs in a developed economy context (Thompson, 2009; Morris et al., 2013), with little scientific evidence from developing economies (Urban, 2013). The value of this study stems from both exploring this relationship in a developing country context as well as on a nascent sample, which has received scant research attention, as most previous studies investigated student samples in their EI research. This benefits the reviewing of the existing theory within a novel research context. This research is applicable to other developing countries with similar entrepreneurial activity levels while maintaining far broader applicability regarding theory development. Educators could take note of the cognitive and functional higher-order competence construct with the individual ECs and include them in entrepreneurship programmes to enhance the action taken by nascent entrepreneurs. In particular, educators should take note of the fact that individual ECs must be developed in conjunction with other ECs that are categorised together. For example, cognitive and functional competence, opportunity recognition; opportunity assessment; conveying a compelling vision; creative problem-solving and value creation should be developed and trained together.

### Limitations and Future Research

No study is without limitations. Firstly, although scholars call for more entrepreneurship research from Africa, our research tests the EI–EC relationships in one particular setting, namely South Africa. Testing these higher-order competence constructs and EI relationships in other contexts and, for example, comparing the results to developed countries could contribute to the generalisability of results. It would be interesting to investigate whether all four higher-order competence constructs with their individual ECs and the relationship with EI would provide an acceptable model fit in a developed country context. Secondly, we acknowledge that different individual ECs might be categorised under each higher-order competence construct and the EI–EC model might be presented differently by other scholars in the field. We call on future research to investigate these higher-order categories and to explore which individual ECs are categorised under each, this should be tested in divergent settings and contexts. Thirdly, we acknowledge that there are a number of other scales dealing with EI and ECs. Future research could expand these findings, by using other scales and other ECs not included in this paper and comparing the results with those of this paper. Fourth, we have tested only the relationship between intention and competencies rather than the relationship with entrepreneurial action. A longitudinal study on this relationship could test entrepreneurs at different stages of the venture life cycle to indeed investigate when entrepreneurial action occurred and/or was enhanced. Finally, future research could include additional variables to investigate the influence of moderators in the relationship between EI and ECs, for example, demographic variables such as age and gender, or other personal factors such as physical characteristics and prior entrepreneurial experience. In this regard, it may be valuable to investigate self-efficacy as a mediator and/or moderator in the relationship between EI and ECs as scholars have suggested that ECs should enhance an entrepreneur's actual and perceived self-efficacy. It is further suggested that self-efficacy is tested in an EI–EC model as higher-order competence constructs with individual ECs such as opportunity recognition and assessment categorised under self-efficacy as the previous literature studies suggest that these constructs are positively related (Schlaegel and Koenig, 2014; Fayolle et al., 2015; Miralles et al., 2016).

## Data Availability Statement

The datasets generated for this study are available on request to the corresponding author.

## Ethics Statement

The studies involving human participants were reviewed and approved by The Faculty of Economic and Management Sciences, Ethics Committee, University of Pretoria. The patients/participants provided their written informed consent to participate in this study.

## Author Contributions

This article was prepared by two authors MB and AT. MB conceptualised the paper and prepared the literature review and findings. AT collected the data and also contributed to the final preparation of this article. All authors contributed to the article and approved the submitted version.

## Conflict of Interest

The authors declare that the research was conducted in the absence of any commercial or financial relationships that could be construed as a potential conflict of interest.

## Publisher's Note

All claims expressed in this article are solely those of the authors and do not necessarily represent those of their affiliated organizations, or those of the publisher, the editors and the reviewers. Any product that may be evaluated in this article, or claim that may be made by its manufacturer, is not guaranteed or endorsed by the publisher.
